# Increasing expression of substance P and calcitonin gene-related peptide in synovial tissue and fluid contribute to the progress of arthritis in developmental dysplasia of the hip

**DOI:** 10.1186/s13075-014-0513-1

**Published:** 2015-01-12

**Authors:** Hui Wang, Xiang Zhang, Ji-Ye He, Xin-Feng Zheng, De Li, Zheng Li, Jun-Feng Zhu, Chao Shen, Gui-Quan Cai, Xiao-Dong Chen

**Affiliations:** Department of Orthopaedic Surgery, Xinhua Hospital, Shanghai Jiaotong University School of Medicine, No. 1665, Kongjiang Road, Yangpu District, Shanghai, 200092 People’s Republic of China

## Abstract

**Introduction:**

Developmental dysplasia of the hip (DDH) is a common musculoskeletal disorder that has pain and loss of joint function as major pathological features. In the present study, we explored the mechanisms of possible involvement and regulation of substance P (SP) and calcitonin gene-related peptide (CGRP) in the pathological and inflammatory processes of arthritis in DDH.

**Methods:**

Blood, synovial tissue and fluid samples were collected from patients diagnosed with different severities of DDH and from patients with femoral neck fracture. Levels of SP, CGRP and inflammatory cytokines in synovium and synovial fluid (SF) in the different groups were evaluated by immunohistochemistry, real-time PCR and enzyme-linked immunosorbent assay (ELISA). Correlations between neuropeptides and inflammatory cytokines in SF were evaluated by partial correlation analysis. The proinflammatory effects of SP and CGRP on synoviocytes obtained from patients with moderate DDH were investigated *in vitro* by real-time PCR and ELISA. The mechanisms of those effects were evaluated by Western blot analysis and nuclear factor κ-light-chain-enhancer of activated B cells (NF-κB) DNA binding assay.

**Results:**

Significantly increased levels of neuropeptides and inflammatory cytokines were observed in synovium and SF from patients in the severe DDH group compared with the moderate DDH and control groups. In moderate DDH samples, SP in SF correlated with tumor necrosis factor (TNF)-α, and CGRP in SF correlated with TNF-α and interleukin (IL)-10. In the severe DDH group, SP in SF correlated with interleukin (IL)-1β, TNF-α and IL-10. CGRP in SF correlated with TNF-α. Additionally, SP might have had obvious proinflammatory effects on synoviocytes through the activation of NF-κB.

**Conclusions:**

The upregulation of SP and CGRP in synovium and SF might participate in the inflammatory process of arthritis in DDH. The activation of the NF-κB pathway seems indispensable in the proinflammatory effect of SP on synoviocytes. This original discovery may indicate a potential clinical drug target and the development of innovative therapies for DDH.

## Introduction

**Figure 1:**
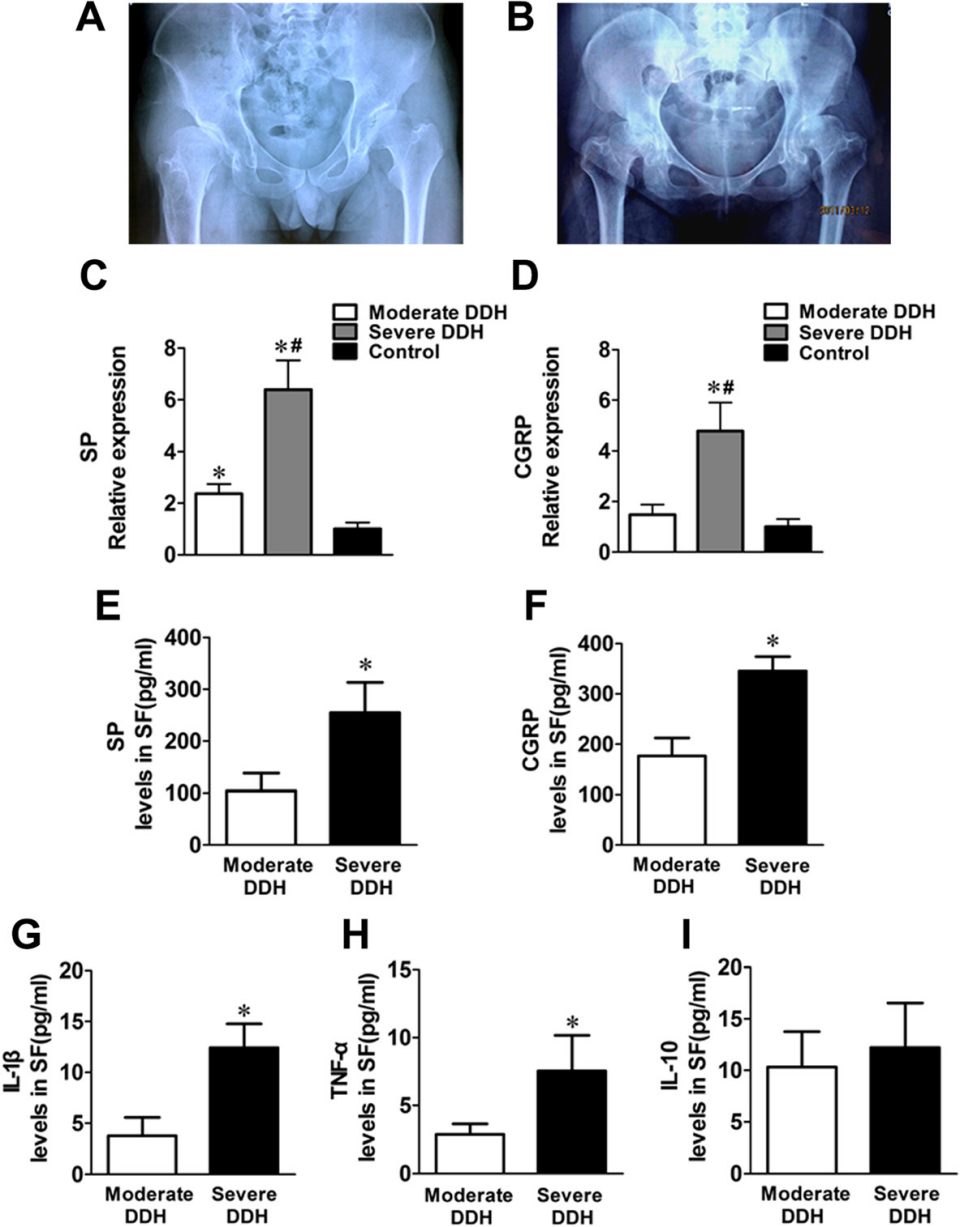
**Radiographs and experimental data of patients with developmental dysplasia of the hip.** Radiographs depicting moderate developmental dysplasia of the hip (DDH) **(A)** and severe DDH **(B)**. mRNA expression of substance P (SP) **(C)** and calcitonin gene-related peptide (CGRP) **(D)** in synovial tissues obtained from patients in the study groups: moderate DDH (*n* = 10), severe DDH (*n* = 11) and control (*n* = 8). *P < 0.05 versus control group. #*P* < 0.05 versus moderate DDH group. **(E)** through **(I)** Concentrations of neuropeptides (SP and CGRP) and inflammatory cytokines (interleukin (IL)-1β, tumor necrosis factor (TNF)-α and IL-10) were detected by enzyme-linked immunosorbent assay. *P < 0.05 versus moderate DDH. SF, Synovial fluid. Data represented are mean ± standard deviation.

Developmental dysplasia of the hip (DDH), formerly known as congenital dysplasia of the hip, affects 1% to 5% of babies in China, and the reported female-to-male ratio ranges from 4:1 to 10:1 in different populations [1,2]. Shallow acetabulum, slacking joint capsule, narrowing joint space and disorder of the hip joint are the main anatomic features of DDH, which result in chronic pain, inflammation around the hip joint, synovial hyperplasia and hypertrophy in fossa acetabuli. Abnormal shear load on the cartilage surface can also induce secondary osteoarthritis (OA) and cartilage degeneration. In addition, a phenomenon frequently observed in young adult patients in the clinic is moderate DDH (slight pain and joint restriction) (Figure 1A) that leads to severe DDH (acute pain, joint dysfunction and obvious OA) (Figure 1B) within a few years. Undoubtedly, both of these stages of DDH can sharply diminish the patient’s quality of life.

Clinically, series of osteotomies have been widely implemented for the treatment of DDH in young adults [3]. However, little is known about the mechanisms of pain and the pathogenesis of arthritis development in DDH. In recent studies, researchers have observed synovial hyperplasia and hypertrophy in the acetabular fossa, especially in weight-bearing areas of the hip joint, in patients with OA [4,5]. Furthermore, sensory nerve fibers in human labrum and synovium have been found to be immunoreactive with protein gene product 9.5, calcitonin gene-related peptide (CGRP) and tumor necrosis factor (TNF)-α in patients with hip pain or rheumatoid arthritis (RA) [6], suggesting the possible involvement and regulation of sensory nerves in the pathogenesis of hip pain.

Inflammatory processes and associated activation of the central and peripheral nervous systems are regarded as the main reasons for the pain sensation in OA. Among these reasons, tachykinin family members, such as substance P (SP) and CGRP, that participate in nociceptive transmission and the proinflammatory process have been proved to be widely involved in the progression of knee and ankle joint arthritis [7,8]. In addition, inhibitors of the ubiquitin–proteasome system, along with other forms of pain-relieving therapies, have been found to be able to restrain the progression of arthritis in rats by specifically decreasing the expression of SP and CGRP in synovium [9,10].

SP is a member of tachykinin family and is known to be extensively involved in the neurogenic inflammatory process [11]. Upregulated SP levels in synovium and synovial fluid (SF) have been observed in patients with RA [12]. In addition, SP has been demonstrated to facilitate vasodilatation and plasma extravasation into the synovial cavity, which probably contributes to arthritis progression [13]. The biological responses to SP are mediated by the neurokinin-1 receptor, a G protein–coupled receptor for SP bearing seven transmembrane domains, which have been observed in lymphocytes, monocytes, macrophages and rheumatoid synovial fibroblasts [14].

CGRP is a 37–amino acid neuropeptide that is released primarily from sensory nerve fibers and is well known as the most potent and longest-lasting microvascular vasodilator *in vitro* and one of the most hypotensive agents *in vivo* [15]. CGRP distributes throughout the central and peripheral nervous systems and exhibits a range of biological effects on the mediation of inflammation and pain. Owing to its potent cardiovascular effects, CGRP, together with SP, is widely involved in the inflammatory pain syndrome of arthritis, as well as in other acute and chronic diseases, such as thermal injury and periodontitis [16,17].

Proinflammatory cytokines also play a pivotal role in the pathophysiological process of RA [18]. A close association between the augmented level of inflammatory cytokines in SF and functional degeneration of articular cartilage has been observed in patients with RA [19,20]. Interleukin (IL)-1β and TNF-α secreted by activated synoviocytes, monocytes and macrophages were observed to induce cascades of inflammatory events, including cartilage degrading matrix metalloproteinase (MMP) expression, prostaglandin E_2_ release and nitric oxygen production [21-24]. These cytokines could act as a trigger, exacerbating the inflammatory process and stimulating the release of chemokines. The inflammatory cascade was observed to intensify as a result, which in turn increased the destruction area [25].

Interactions between neuropeptides and cytokines in RA and OA are well recognized. However, the research to date regarding the mechanism of pain development and the pathological progress of arthritis in DDH is limited. In addition, the relationship between neuropeptides and proinflammatory cytokines in the synovium and SF of patients with different stages of DDH remains unknown. Therefore, the objective of this study was to explore the possible proinflammatory properties of neuropeptides in the synovium and SF of patients with DDH and to investigate the role played by neuropeptides during the development of DDH. We sought to improve the understanding of the pathological process in DDH.

## Methods

### Patients and materials

This study was carried out in accordance with the Declaration of Helsinki and was approved by the ethics committee of Xinhua Hospital affiliated to Shanghai Jiao Tong University School of Medicine. Samples obtained from operations were prepared only for testing purposes and with the approval and signed consent of the patients.

The clinical data and samples included in this study were obtained from patients who underwent surgical treatment at our institution between March 2012 and December 2013. Patients involved in this research were divided into three groups according to their clinical diagnosis and disease severity. Thirty-five patients (mean age, 23.8 years; age range, 21 to 36 years) with DDH (Crowe grades I and II) and OA (grade 0 or 1 on the Kellgren-Lawrence (K-L) scale) were set as the moderate DDH group. Thirty-two patients (mean age, 27.4 years; age range, 26 to 46 years) with DDH (Crowe grade I or II) and OA (grade 3 or 4 on the K-L scale) were set as the severe DDH group. The degree of hip joint dislocation and severity of OA were divided according to the patients’ Crowe and K-L grades, respectively, which were determined in the clinic [26,27]. The reference group consisted of 15 patients (mean age, 30.4 years; age range, 25 to 38 years) who underwent traumatic femoral head or neck fracture but had no joint diseases noted in their previous medical records [28-30].

The diagnosis of DDH was based on the standard of a hip with a sharp angle <45° and a center edge angle <20° [31,32]. Patients with moderate DDH underwent periacetabular osteotomy (PAO) or osteochondroplasty (OCP), and those with severe DDH underwent total hip arthroplasty (THA). Patients in the control group underwent emergency internal fixation or hemiarthroplasty within 24 hours after injury. The data compiled from clinical and laboratory examinations of patients in all groups are listed in Table 1.

**Table 1:** **Clinical information of patients recruited into this study** ^**a**^

	**Moderate DDH**	**Severe DDH**	**Control**
Number of patients	35	32	15
Mean age (yr)	23.8 ± 6.5	27.4 ± 5.7	30.4 ± 3.5
Female/male (*n*)	28/7	22/10	9/6
BMI (kg/m^2^)	23.4 ± 2.1	24.8 ± 2.3	23.8 ± 1.9
Duration (yr)	2.7	5.5	–
CRP (mg/L)	11.79 ± 3.8	13.1 ± 4.6	10.9 ± 6.5
ESR (mm/1st hr)	16.2 ± 3.0	24.4 ± 4.5*	19.6 ± 4.7
Harris Hip Score	74.6 ± 9.9	62.8 ± 12.4*	–
Visual analogue scale score	38.2 ± 13.2	57.8 ± 10.3*	–

^a^BMI, Body mass index; CRP, C reactive protein; DDH, Developmental dysplasia of the hip; ESR, Erythrocyte sedimentation rate. The Mann–Whitney *U* test was applied for comparisons between groups. *P < 0.05 versus moderate DDH group. Data are presented as mean ± standard deviation.

The synovial tissues obtained during the operations were immediately cut into 1-cm^3^ pieces and prepared for three applications. (1) Some pieces were immerged into RNA*later* (catalog number AM7021; Ambion, Austin, TX, USA) overnight and then stored at −80°C for RNA extraction. (2) Some specimens were immersed in 4% paraformaldehyde for 24 hours, washed, dehydrated with a graded series of alcohol solutions and finally embedded in paraffin for immunohistochemical (IHC) analysis. (3) The rest of the sections were immediately stored at −80°C for Western blot analysis.

### Detection and analysis of substance P and CGRP in hip joint synovial tissue by immunohistochemistry

Hematoxylin and eosin (H-E) (Sigma-Aldrich, St Louis, MO, USA) staining and IHC staining for SP and CGRP were performed. After citrate antigen retrieval and peroxidase inactivation, the synovial slides in each group were incubated overnight with the primary antibodies to SP (1:1,000 dilution, catalog number ab14184; Abcam, Cambridge, UK) and CGRP (1:3,000 dilution, catalog number ab36001; Abcam). After that, slides were incubated with biotinylated secondary antibody (EnVision system; Dako, Glostrup, Denmark) for 30 minutes. Finally, 3′-diaminobenzidine tetrachloride was used for visualization of immunoreactions. From every synovium sample (six per group), two sections were acquired at different depths, and eight microscopic fields from each section were photographed and analyzed. Immunoreactivity was measured as the area in square millimeters using Image-Pro Plus version 6.0 software (Media Cybernetics, Rockville, MD, USA). The results are expressed in the form of mean optical density, which we calculated as the integral optical density value of each microscopic field in relation to the actual synovial area.

### Gene expression detection of substance P and CGRP in synovium of hip joint by real-time PCR

Synovial tissues from fossa acetabuli were obtained during THA or PAO, as described above, from 10 patients with moderate DDH, 11 with severe DDH and 8 in the control group. The sequences of primers used to amplify mRNA are shown in Table 2. Real-time PCR was carried out by using 200 ng of cDNA and the PrimeScript RT-PCR Kit (catalog number DRR064A; Takara, Ōtsu, Japan) in 96-well plates in an ABI 7500 Sequence Detection System (Applied Biosystems, Foster City, CA, USA) according to the manufacturer’s instructions. Relative quantification of each gene was normalized to the housekeeping gene *β-actin*. Relative mRNA expression levels of neuropeptides were calculated by using the cycle threshold method [33]. Analysis of the results is based on at least three individual experiments.

**Table 2:** **Sequences of PCR primers related to neuropeptides and inflammatory cytokines included in this study** ^**a**^

**Gene**	**Forward primer (5′-3′)**	**Reverse primer (5′-3′)**
*TAC1* (SP)	GGTACGACAGCGACCAGATCA	CCCGTTTGCCCATTAATCCA
*CGRP*	GCAAGAGAGAGAGGGCTCCA	TTTCTTTCCAGGTGCTCCAA
*IL-1β*	CCTTGTCCTGCGTGTTGAAAGA	AAACTCAGACGGGTCAAGGG
*IL-6*	GATGAGTACAAAAGTCCTGATC	CTGCAGCCACTGGTTCTGT
*TNF-α*	CAGCCTCTTCTCCTTCCTGAT	GCCAGAGGGCTGATTAGAGA
*MMP-13*	CCCTTGATGCCATTACCAGTC	TCCGCATCAACCTGCTGAG
*MMP-3*	TGATGAACAATGGACAAAGGATAC	CTGTGAGTGAGTGATAGAGTGG
*TIMP-1*	TGCCGCATCGCCGAGAT	ATGGTGGGTTCTCTGGTG
*β-actin*	CATCAAGAAGGTGGTGAAGCAG	TGTAGCCAAATTCGTTGTCATACC

^a^CGRP, Calcitonin gene-related peptide; IL, Interleukin; MMP, Matrix metalloproteinase; TIMP, Tissue inhibitor of metalloproteinase; TNF, Tumor necrosis factor.

### Enzyme-linked immunosorbent assay of neuropeptides and cytokines in synovial fluid

Concentrations of TNF-α, IL-1β and IL-10 in SF were detected by using human enzyme-linked immunosorbent assay (ELISA) kits (catalog numbers EHC103a, EHC002b and EHC009 (H), respectively; Xinbo Sheng, Shenzhen, China) in accordance with the protocol provided by the manufacturer. Levels of SP and CGRP in SF were measured with human ELISA kits (catalog numbers 589101 and 583751, respectively; Cayman Chemical, Ann Arbor, MI, USA) according to the manufacturer’s instructions.

### Incubation of human fibroblast-like synoviocytes

Fibroblast-like synoviocytes (FLSs) were isolated from the hip joint synovium of patients with moderate DDH during their PAO and OCP procedures. Synovial samples were sheared into pieces 1 × 1 mm^3^ in size and repeatedly washed with phosphate-buffered saline (PBS) and then digested with collagenase I (1 mg/ml; Sigma-Aldrich) and 0.25% trypsin-ethylenediaminetetraacetic acid (catalog number 25300–054; Gibco/Life Technologies, Grand Island, NY, USA) for 3 hours and 1 hour, respectively, at 37°C. The cell suspension was filtered through a cell strainer (80 mm) and washed with PBS. After centrifugation, cells were washed and seeded in Dulbecco’s modified eagle medium/F-12 medium with 10% fetal calf serum (FCS), 100 μg/ml penicillin and 100 U/ml streptomycin at 37°C, 95% air humidity and 5% CO_2_. Nonadherent cells were discarded after overnight incubation. Cells were plated at a density of 4 × 10^5^ cells/dish in a six-well plate. Cells at the third to sixth passages, which were recognized as mature human synoviocytes (>95% FLS purity), were prepared for further treatment [34,35].

### Gene expression of inflammatory cytokines in cultured fibroblast-like synoviocytes after treatment with substance P or CGRP

We did a concentration gradient study by ELISA to obtain the most suitable neuropeptide concentration according to the production of inflammatory cytokines (IL-1β, TNF-α). After 24-hour serum starvation (2% FCS), the neuropeptides SP (catalog number S6883; Sigma-Aldrich) and CGRP (catalog number C0167; Sigma-Aldrich) were applied for 24 and 48 hours, respectively, as a time course study.

The relative expression of genes related to inflammation and cartilage degeneration, including IL-1β, TNF-α, IL-6, MMP-13, MMP-3 and tissue inhibitor of metalloproteinase (TIMP)-1, was detected by using a real-time PCR detection system. The results were normalized against β-actin and control conditions. Three replicates were performed, and the results were averaged.

### Western blot analysis

Nuclear cell extracts were collected with a kit manufactured by Active Motif (Carlsbad, CA, USA). Nucleus protein lysates (30 μg) were separated by using 10% SDS-PAGE gel and transferred onto a nitrocellulose membrane. The membrane was blocked with 5% fat-free dry milk and then incubated overnight at 4°C with a 1:1,000 dilution of an anti-human phosphorylated and nonphosphorylated p65 antibody (catalog number 4767; Cell Signaling Technology, Danvers, MA, USA). After being washed, the membrane was incubated with a 1:1,000 dilution of a peroxidase-conjugated anti-rabbit secondary antibody (catalog number 7074; Cell Signaling Technology) at room temperature for 2 hours. Detection was achieved with the help of an enhanced chemiluminescence kit (EMD Millipore, Billerica, MA, USA). The Western blotting signals were measured by scanning densitometry and normalized against β-actin (catalog number 4967; Cell Signaling Technology).

### NF-κB activation assay

The activation of NF-κB binding to DNA was measured in nuclear extracts from synoviocytes pretreated with SP, CGRP and their inhibitors L-703606 (Sigma-Aldrich) and MK-3207 (Selleck Chemicals, Houston, TX, USA) with an ELISA-based TransAM NF-κB p65 assay kit (Active Motif). The NF-κB complex binding to the oligonucleotide was detected by spectrophotometry with the use of a secondary antibody conjugated to horseradish peroxidase. The results are presented as activation fold changes with the unstimulated control set to 100%.

### Enzyme-linked immunosorbent assay for role of NF-κB in proinflammatory effect of substance P and CGRP on synovial cells

Western blot analysis and NF-κB DNA binding assays were carried out to select the effective concentration of NF-κB inhibitor Bay 11–7085 (Sigma-Aldrich), which was preincubated with synovial cells at 1 μM, 10 μM or 30 μM for 2 hours, followed by treatment with SP (100 nM) or CGRP (1 nM). To evaluate the contribution of NF-κB in the secretion of cytokines, the synovial cells were preincubated with L-703606 (10 μM), MK-3207 (1 μM) or Bay 11–7085 (30 μM) for 2 hours, followed by stimulation with SP (100 nM) or CGRP (1 nM) for 24 hours. Supernatants were collected and prepared for analysis in which we used ELISA kits specific for IL-1β and TNF-α.

### Criteria for exclusion

Patients recruited into this study had no secondary OA as a consequence of rheumatism, trauma, gout or tumor. Patients with DDH secondary to slipped capital femoral epiphysis or traumatic OA, as well as those who underwent orthopedic surgery in the hip, were also excluded.

### Statistics

The results are expressed as mean ± standard deviation. Statistical analyses were performed using SPSS statistical software (SPSS, Chicago, IL, USA). Comparisons of SP and CGRP levels in the synovium and SF in these groups were carried out by using ELISA, immunohistochemistry and real-time PCR; Student’s Mann–Whitney *U* test, a two-tailed *t*-test and analysis of variance; and GraphPad Prism version 5.01 software (GraphPad Software, La Jolla, USA). *P*-values <0.05 were considered significant. After adjustment for confounding factors, correlation analyses were performed with Spearman’s rank correlation test. A two-sided *P*-value <0.05 was considered statistically significant.

## Results

### Increased vascularization and neuropeptide expression in synovium and synovial fluid of patients with developmental dysplasia of hip

**Figure 2:**
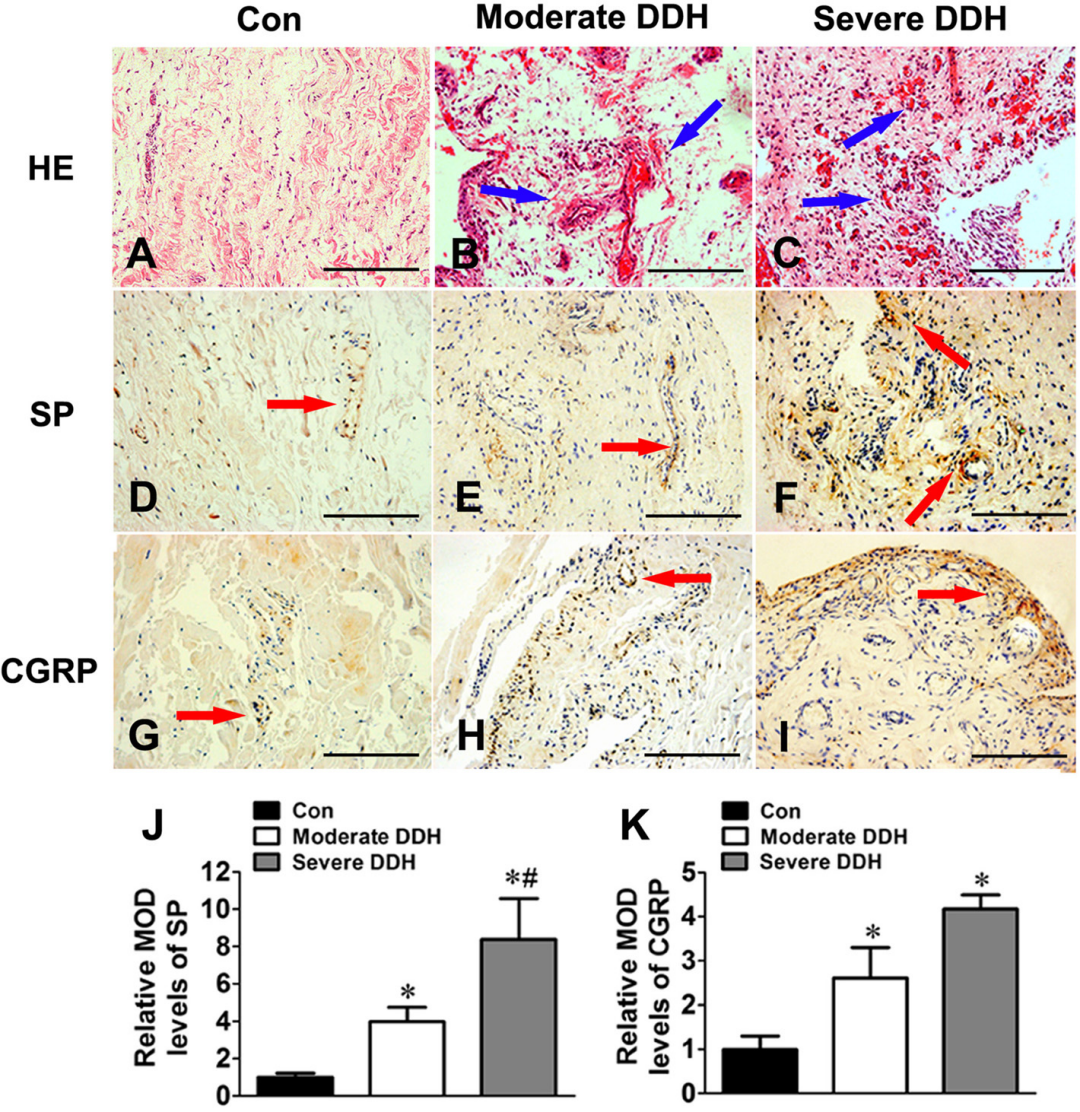
**Photomicrographs showing staining of substance P and calcitonin gene-related peptide from synovial tissues among different groups. (A)** Hematoxylin and eosin (HE) stain from the control sample. Blue arrows in **(B)** and **(C)** represent vessels or hyperplastic capillaries in synovial tissues (HE staining). Red arrows in **(D)** through **(I)** indicate positive immunohistochemical staining of substance P (SP; **(D)**, **(E)** and **(F)**) or calcitonin gene-related peptide (CGRP; **(G)**, **(H)** and **(I)**). Scale bars = 100 μm. **(J)** and **(K)** Graphs depict mean optical density values of the positive immunohistochemical staining of SP and CGRP separately in each group. Data presented are mean ± standard deviation. *P < 0.05 versus control. #*P* < 0.05 versus moderate developmental dysplasia of the hip (DDH).

During the progression from moderate to severe DDH, the hip joint synovium showed much more extensive vascularization and inflammatory cell infiltration, which were detected by H-E staining (Figure 2A-C). IHC staining (Figure 2D-I) and gray staining (Figure 2 J,K) analyses showed stronger staining of SP and CGRP in patients with severe DDH than in those with moderate DDH and those in the control group. Analogous results were also observed in the comparison of neuropeptide gene expression in the synovial tissues of these groups (Figure 1C,D). Furthermore, levels of SP and CGRP in the SF of patients with severe DDH increased significantly compared to levels in patients with moderate DDH (Figure 1E,F). Additionally, in comparisons between the moderate and severe DDH groups regarding inflammatory cytokines in SF, we found statistically significant differences for IL-1β (*P* < 0.05) and TNF-α (*P* < 0.05) (Figure 1G,H).

### Correlations between neuropeptides and cytokines in synovial fluid

The results shown in Table 3 indicate that, in moderate DDH cases, the concentration of SP in SF correlated with TNF-α (*r*_S_ = 0.41, *P* < 0.05) and CGRP in SF correlated with IL-10 (*r*_S_ = 0.69, *P* < 0.01) and TNF-α (*r*_S_ = 0.40, *P* < 0.05). In severe DDH cases, the concentration of SP in SF correlated with IL-1β (*r*_S_ = 0.45, *P* < 0.05) and TNF-α (*r*_S_ = 0.48, *P* < 0.05) and IL-10 (*r*_S_ = 0.39, *P* < 0.05). Levels of CGRP in SF correlated with TNF-α (*r*_S_ = 0.47, *P* < 0.01).

**Table 3:** **Correlation of substance P and CGRP with IL-1β, TNF-α and IL-10 in synovial fluid of patients with developmental dysplasia of the hip** ^**a**^

		**Correlation of SF neuropeptide levels**					
**Group**	**Neuropeptide**	**With IL-1β**		**With TNF-α**		**With IL-10**	
Moderate DDH		R	*P*-value	R	*P*-value	R	*P*-value
	SP	0.19	0.30	0.41	<0.05*	0.33	0.08
	CGRP	0.10	0.64	0.40	<0.05*	0.69	<0.01*
Severe DDH							
	SP	0.45	<0.05*	0.48	<0.05*	0.39	<0.05*
	CGRP	0.37	0.07	0.47	<0.01*	0.14	0.46

^a^CGRP, Calcitonin gene-related peptide; DDH, Developmental dysplasia of the hip; IL, Interleukin; R, Correlation index; SF, Synovial fluid; SP, Substance P; TNF, Tumor necrosis factor. *A two-sided *P*-value <0.05 indicates a significant correlation with the corresponding neuropeptide in each column.

### Increased gene expression of inflammatory cytokines in substance P- and CGRP-treated human synovial cells

**Figure 3:**
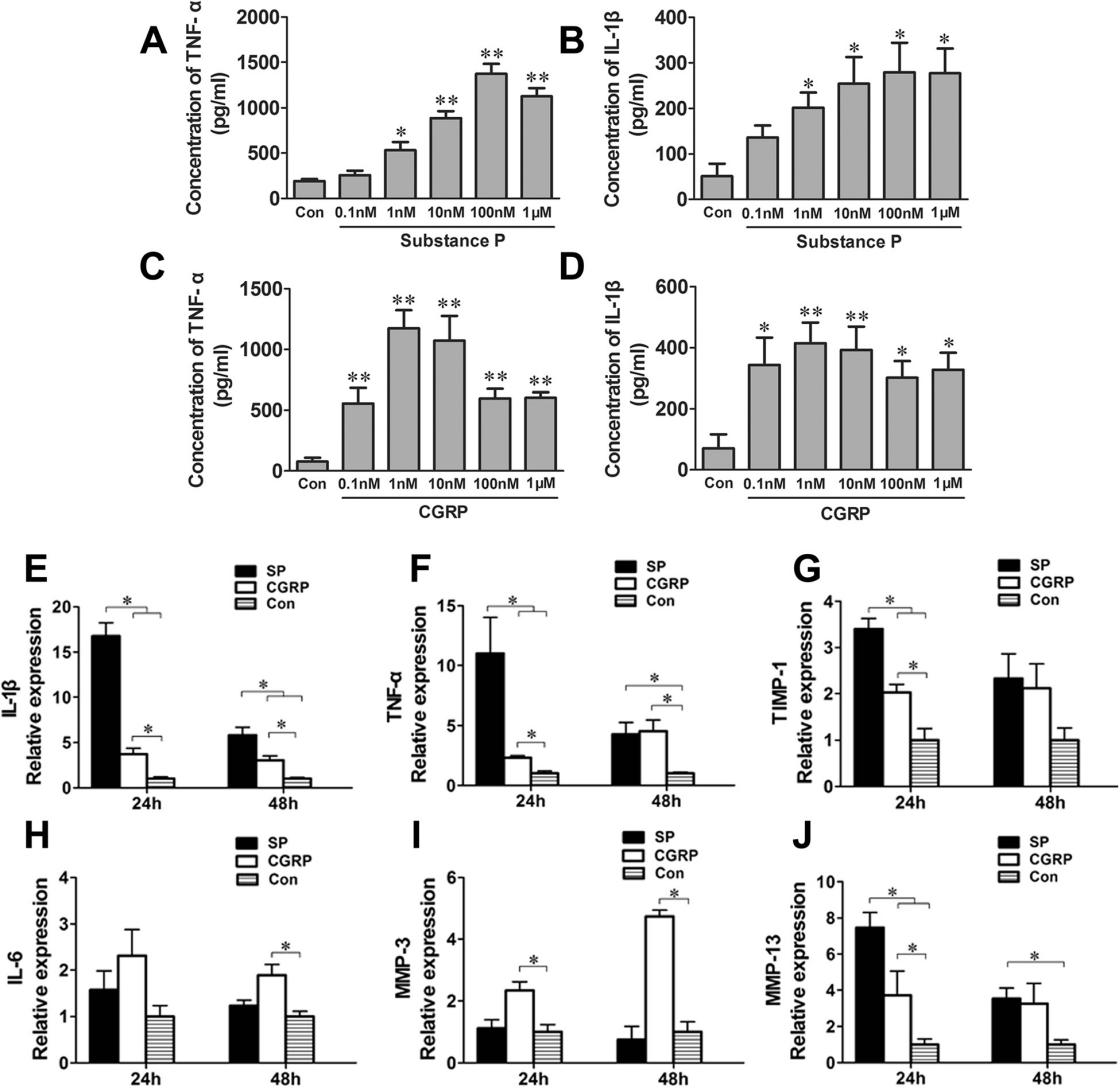
**The proinflammatory effect of neuropeptides on synoviocytes** ***in vitro*** **. (A-D)** Dose dependence of substance P (SP)- and calcitonin gene-related peptide (CGRP)-induced tumor necrosis factor (TNF)-α and interleukin (IL)-1β production. *P < 0.05 and **P < 0.01 versus control. **(E-J)** Genes for inflammation-related cytokines and cartilage erosion factors were evaluated in cultured fibroblast-like synoviocytes after 24 or 48 hours of treatment with SP or CGRP, respectively. MMP, Matrix metalloproteinase; TIMP, Tissue inhibitor of metalloproteinase. The error bars indicate the standard deviation. *P < 0.05. Three replicates were performed, and the results were averaged.

The results of the concentration gradient study showed that 100 nM SP and 1 nM CGRP had the strongest proinflammatory effect (Figure 3A-D). After 24-hour treatment, greater gene expression of inflammation-related cytokines (IL-1β, TNF-α) and collagen metabolism-related factors (TIMP-1 and MMP-13) were observed in synoviocytes with SP compared to the unstimulated group. The trend weakened with time. In addition, CGRP could upregulate the expression of these cytokines by 2 days of culture, which might indicate a delayed inflammatory effect (Figure 3E-J).

### Effects of substance P and CGRP in activation of NF-κB pathway in synovial cells

**Figure 4:**
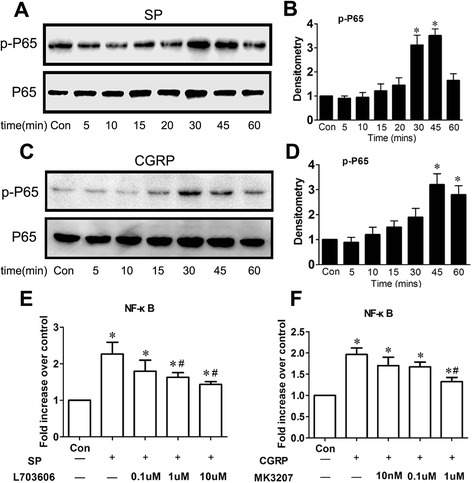
**Activation of NF-κB pathway evaluated by Western blot analysis and NF-κB DNA binding assay. (A-D)** Synoviocytes obtained from patients with developmental dysplasia of the hip (DDH) were incubated with 100 nM substance P (SP) or 1 nM calcitonin gene-related peptide (CGRP) within 1 hour. Nuclei cell proteins were subjected to Western blot analysis **(A)** and **(C)** with antibodies against phosphorylated p65 (p-P65). **(B)** and **(D)** Densitometric analysis of Western blotting data derived from **(A)** and **(C)**. *P < 0.05 versus control. **(E)** and **(F)** Nuclear factor κ-light-chain-enhancer of activated B cells (NF-κB) p65 and DNA binding assays. Synoviocytes were pretreated with L-703606 or MK-3207 for 2 hours, followed by 1 hour of stimulation with SP (100 nM) or CGRP (1 nM). *P < 0.05 versus control. #*P* < 0.05 versus synoviocytes stimulated with SP or CGRP alone.

As shown in Figure 4A and 4B, SP could stimulate the activation of phosphorylated p65 (phospho-p65) in synovial cells. The activation was evident at 30 minutes and increased up to 45 minutes. Analogously, as shown in Figure 4C and 4D, CGRP could also induce an increase of phospho-p65 at 45 minutes compared to control.

NF-κB p65 DNA binding assays in (Figure 4E,F) revealed that treatment with either SP or CGRP could lead to a notable increase in the activity of NF-κB p65 and that this effect could be blocked by the inhibitors of these neuropeptides (L-703606 for SP and MK-3207 for CGRP).

### Effect of NF-κB inhibitors on secretion of inflammatory cytokines in synovial cells

**Figure 5:**
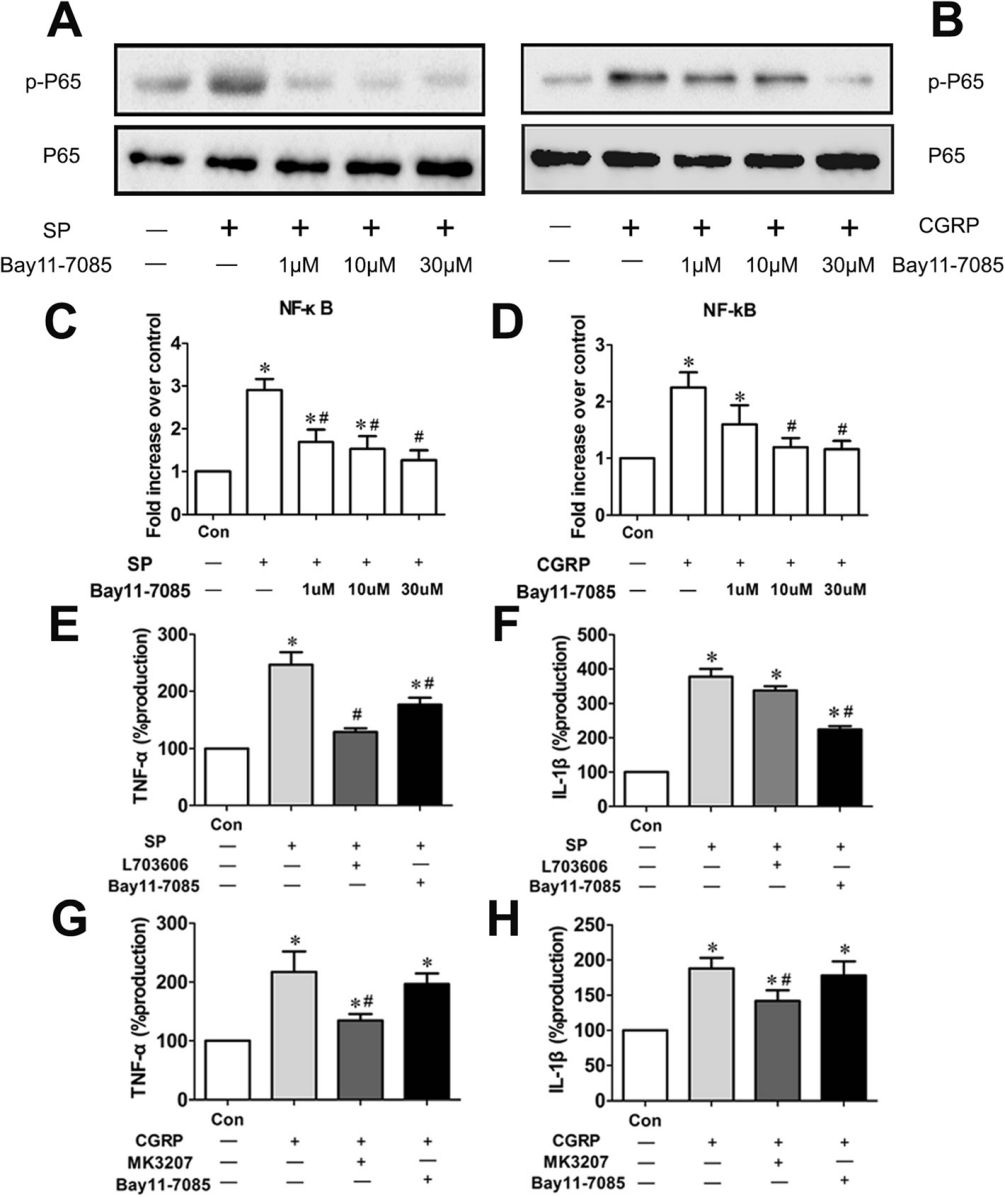
**The role of the NF-κB pathway played in the proinflammatory effect of substance P and calcitonin gene-related peptide on synovial cells. (A-D)** Western blot and DNA binding assays were carried out for the selection of effective concentration of Bay 11–7085. CGRP, Calcitonin gene-related peptide; NF-κB, Nuclear factor κ-light-chain-enhancer of activated B cells; p-P65, Phosphorylated p65; SP, Substance P. **(E-H)** Supernatants were collected from each group and prepared for enzyme-linked immunosorbent assay kits specific for interleukin (IL)-1β and tumor necrosis factor (TNF)-α. Mean production (in picograms per milliliter (pg/ml)) of IL-1β and TNF-α in unstimulated synovial cells was used as a control (100% production). IL-1β and TNF-α production (pg/ml) by the other incubation cultures are expressed as the percentage of control. *P < 0.05 versus control. #*P* < 0.05 versus stimulation with SP or CGRP alone. Three replicates were performed, and the results were averaged. Data are represented as mean ± standard deviation.

Western blot and NF-κB DNA binding assays (Figure 5A-D) showed that Bay 11–7085 (30 μM) could effectively block the activation of the NF-κB pathway. Treatment of synoviocytes with 100 nM SP or 1 nM CGRP led to obvious upregulated secretion of IL-1β and TNF-α compared to unstimulated cells. Bay 11–7085 (30 μM) could significantly block the proinflammatory effect of SP on synoviocytes (Figure 5E,F), but the proinflammatory effect of CGRP could not be inhibited in the presence of Bay 11–7085 (Figure 5G,H).

## Discussion

Because our department is the largest DDH center in China (with more than 300 young patients diagnosed with DDH every year), it provides convenience for acquiring precious synovial tissues and fluid samples during surgery. This is the first time that the histological characteristics of synovial tissue were obtained from a diverse sample of patients with moderate or severe DDH. We are probably the pioneers in incubating synoviocytes from patients with moderate DDH in *in vitro* experiments and the first to investigate the proinflammatory effects of SP and CGRP on synoviocytes. In addition, for the first time, upregulated expression of neuropeptides in the hypertrophic synovial tissue of patients with DDH was detected by IHC staining. We know that pain signal neuropeptides (SP and CGRP) are synthesized mainly in sympathetic ganglia and transferred and secreted by peripheral sympathetic fibers. Our observations in this study suggest that more sympathetic fibers might migrate and distribute into the hip joint with the development of DDH. These nerve fibers might also engage in the transmission of pain sensation in the hip joint, which helps us better understand the mechanisms of pain creation and the progression of inflammation in different stages of DDH.

In this study, we divided patients with DDH into two groups (moderate DDH and severe DDH). By doing so, we aimed to investigate the differences in pathophysiological processes and inflammatory conditions between these groups and to identify the role of SP and CGRP in the progression from moderate to severe DDH. The results of immunohistochemistry, real-time PCR and ELISA experiments demonstrated remarkable upregulation of SP and CGRP in both synovium and SF obtained from patients with severe DDH compared with those from patients with moderate DDH. Previous studies demonstrated that, with the stimulation of peripheral pain, SP and CGRP were increasingly synthesized in dorsal root ganglia (DRG), then secreted by peripheral sensory nerve endings [36]. On the basis of our findings, we speculate that, with the time and duration of DDH, the degree of cartilage wearing gradually increases and the cushioning capacity of cartilage progressively disappears. If that is true, the subchondral bone and labrum tissue will bear increasing shear and compressive loading as cartilage destruction evolves. In this process, the afferent stimulation of joint pain is gradually increased, which can in turn induce greater infiltration of peripheral nerve fibers with increased release of neuropeptides from DRG. In this way, the progression and duration of DDH might stimulate the release of SP and CGRP from nerve fibers in synovial tissue.

In the IHC staining and ELISA experiments, we detected increasing CGRP levels in both synovium and SF with the progression of DDH. We also observed significant proinflammatory capacities of CGRP by real-time PCR (Figure 4) and correlation between CGRP and cytokines (IL-1β, TNF-α and IL-10) in the SF of patients with moderate or severe DDH (Table 3). A previous study proved that CGRP neurons play crucial roles in the perception of pain in the hip joint [37-39]. Collectively, our results favor the hypothesis that CGRP might be involved in not only the hyperalgesia but also the regulation of inflammatory infiltration in the hip joints of patients with DDH, which probably have an impact on the pathological process of DDH.

Similarly, we also observed the upregulation of SP levels in synovial tissues obtained from patients with different stages of DDH compared with those obtained from the control group. It has been reported that SP is involved in the mediation of inflammatory hyperalgesia and immune regulation [40]. In this study, we explored the possible correlation between SP and inflammatory cytokines in the SF of patients with severe DDH. We also investigated the proinflammatory effects of SP in real-time PCR and ELISA experiments, which can be considered direct evidence of SP’s involvement in inducing inflammatory infiltration and the severity of DDH that develops.

In Western blot analysis and NF-κB DNA binding assays, we witnessed the obvious activation of phospho-p65 protein after stimulation with SP or CGRP. The activation of NF-κB, the principal transcriptional regulator of inflammation, was previously demonstrated to be upregulated in synovial and cartilage samples in several animal models of arthritis [41,42]. By the same token, the proinflammatory effect of SP and CGRP associated with the activation of NF-κB pathway has been proved through the culture of synovial cells *in vitro*. Furthermore, when blocked by the inhibitor of NF-κB, the inflammation-promoting effect of SP on synovial cells was obviously restrained (Figure 5E,F). However, suppressing the role of NF-κB in CGRP-pretreated synovial cells could not effectively diminish the release of inflammatory mediators (Figure 5G,H). Thus, it appears that the activation of NF-κB might not crucially mediate or be involved in the release of inflammatory cytokines induced by CGRP.

Though previous studies proved that both SP and CGRP have strong vasodilatation and inflammation chemotactic abilities, there are also some theories that neuropeptides may have diverse roles in the inflammatory process [43,44]. Yuko *et al*., for example, reported that CGRP could inhibit the synoviocyte proliferation and cytokine-induced IL-6, IL-8 and MMP-2 release of synoviocytes from patients with RA [45]. However, in our study, we observed proinflammatory effects of CGRP on synoviocytes from patients with DDH. The exact difference in the role of CGRP as it relates to synoviocytes between patients with RA and patients with DDH is not clear. In addition to the different inflammation sources, which could affect the functional reaction of these two cells, the diverse treatment time and the environment could explain the discrepancy in the inflammatory capabilities of the same neuropeptide in the different settings.

Our study has some limitations. First, owing to medical ethical requirements, it is impossible to obtain perfectly normal synovial specimens. Referring to previous studies on hip joint disease [28-30], we chose patients with femoral head or neck fracture as a control group. Second, as we conducted a kind of phenomenal observational research, the number of patients recruited into each group was not great enough to draw firm conclusions from our results; more tissue samples would have been needed. Additionally, the synoviocytes cultured *in vitro* were obtained from the synovium of patients with moderate DDH, but not from patients with severe DDH. We dedicated our study to exploring novel nonoperative measures to prevent arthritis progression in the primary stage of DDH (moderate DDH), which probably makes more sense than that preventing it in the late stage of DDH (severe DDH). Thus, our results are more targeted on the pathological characteristics of synovium from patients with moderate DDH.

## Conclusions

In general, we observed that SP and CGRP might be involved in the initiation and progression of inflammation in patients with DDH. These findings may help to provide clinical guidelines for novel treatment of patients with DDH. For example, synovectomy, nerve block surgery and intra-articular injection of neuropeptide pathway blockers could be meaningful measures to relieve pain sensation and to slow down the progression of arthritis in patients with DDH.

## Abbreviations

CGRP: Calcitonin gene-related peptide
CRP: C-reactive protein
DDH: Developmental dysplasia of the hip
DRG: Dorsal root ganglion
ELISA: Enzyme-linked immunosorbent assay
ESR: Erythrocyte sedimentation rate
FCS: Fetal calf serum
FLS: Fibroblast-like synoviocyte
IHC: Immunohistochemical
IL: Interleukin
K-L: Kellgren-Lawrence grading system
MMP: Matrix metalloproteinase
NF-κB: Nuclear factor κ-light-chain-enhancer of activated B cells
OA: Osteoarthritis
OCP: Osteochondroplasty
PAO: Periacetabular osteotomy
PBS: Phosphate-buffered saline
RA: Rheumatoid arthritis
SF: Synovial fluid
SP: Substance P
THA: Total hip arthroplasty
TIMP: Tissue inhibitor of metalloproteinase
TNF: Tumor necrosis factor
